# Adolescents' Utilization of a Tertiary-Level Pediatric Emergency Department in Italy

**DOI:** 10.3389/fped.2022.750403

**Published:** 2022-03-10

**Authors:** Giorgio Cozzi, Lisa Passaglia, Anna Agrusti, Manuela Giangreco, Rita Giorgi, Egidio Barbi

**Affiliations:** ^1^Institute for Maternal and Child Health IRCCS Burlo Garofolo, Trieste, Italy; ^2^University of Trieste, Trieste, Italy

**Keywords:** adolescence, pediatric emergency department, mental health, children, utilization

## Abstract

**Aim:**

Describe the use of the emergency department of a tertiary-level children's hospital in Italy by adolescents.

**Methods:**

This retrospective study was based on the medical records of patients aged 13 to 17 years, who accessed the emergency department of the Institute for Maternal and Child Health of Trieste, from 1 January to 31 December 2018. The primary outcome was to describe the leading causes of access, diagnoses, rate of hospitalization, and ward of destination among adolescent patients.

**Results:**

During the study period, 24,599 patients accessed the department. Among them, 3,062 were adolescents, for a total of 3,895 unscheduled visits. The principal causes of access were trauma (45.3%) and organic diseases (38.8%). Two hundred and forty nine adolescents (6.4%) had mental health problems. One hundred and forty two adolescents (3.6%) Were Admitted to the Hospital, 54 of Whom (38%) to the Neuropsychiatric Ward, for Mental Health Problems.

**Conclusions:**

Among adolescents seen in this Italian tertiary-level children's hospital, mental health problems represented a small proportion of emergency department visits but were the leading cause of urgent hospitalization.

## Introduction

Adolescence represents a particular moment in life, characterized by the need to experiment and discover. It is a crucial age for the construction of each person's identity. In a simplified view, the biological basis of adolescence neurocognitive development is underpinned by the maturation of the prefrontal cortex, the region of the brain regulating the rational control of the events. During adolescence, the experience induces mechanisms of pruning and remodeling neuronal connections so that the cortical plasticity typical of this age makes adolescents more vulnerable to the social demands. Overall, the interaction between these mechanisms and the pressure of the society can have substantial psychological consequences (1, 2). Adolescents experience an increase in risk behaviors, associated with a higher incidence of major trauma and delayed access to medical services (3–5). Concern about adolescent mental health has grown in recent years: current studies report a fivefold increase in student's mental health disorders, particularly among young women, with addiction to social media “judgement,” which plays a leading role in focus on psychological stress and social isolation (6). Few studies have been published on adolescent use of emergency services (7, 8) and in detail in the Italian population, in contrast to the growing concern for this age group's wellbeing (1).

This study aimed to describe the use of the pediatric emergency department (PED) of a tertiary-level children's hospital in Italy by adolescents.

## Methods

This retrospective study involved patients evaluated at the PED of the Institute for Maternal and Child Health IRCCS Burlo Garofolo (Trieste, Italy), a tertiary level, University teaching, children's hospital. Trieste is a city of 200.000 inhabitants located in the North East of Italy. Our PED is the only one in town, and has an annual census of 25.000 patients, from zero to 17 years of age.

The study protocol was approved by the Institutional Review Board of the Institute (RC25/2020).

We collected data from the electronic database of the PED, considering the medical records of patients aged 13 to 17 years of age, from 1 January to 31 December 2018. We excluded the records regarding a scheduled re-visit. We considered as scheduled re-visits all patients instructed to come back to the PED to complete the diagnostic work-up or to have a clinical re-evaluation. For each patient, we reported age, gender, date of the visit, nursing triage category, patient's chief complaint, discharge diagnosis, admission status, and hospital ward of destination. In case of overlapping or presence of more symptoms, patients were included in one category according to the findings at the visit and to the PED diagnosis.

Pediatric nurses, with at least 5 years of experience, performed the triage using the Italian National Triage Category system, which consists of four priority levels with increasing severity: white (not-urgent), green (minor emergencies), yellow (urgent) and red (emergency/resuscitation).

According to the main characteristics reported at the visit, we arbitrarily classified the diagnosis into five main domains: trauma, organic diseases, functional diseases, mental health problems, and others. Functional diseases included conditions which fulfilled specific criteria for a defined functional syndrome (9, 10) or patients whose medical history, clinical examination, test results and response to pharmacological therapies were not consistent with an either organic, traumatic or psychiatric condition.

We compared data about triage codes, rate of hospitalization and ward of destination of adolescents with data collected from the population of children (0–12 yrs of age) seen in our PED in the same period of time.

The study's primary outcome was to describe the use of the PED by adolescents in terms of causes of access, diagnosis and rate of hospitalization.

The secondary endpoint was to compare the rate of hospitalization and the ward of destination between adolescents and children accessing the PED.

### Statistical Analysis

The continuous variables were described as median and interquartile ranges, while absolute frequency and percentage were used to describe categorical variables. To evaluate the associations as described in the secondary endpoint, Chi-square test was employed. A *p*-value < 0.05 was considered significant.

## Results

From 1 January to 31 December 2018, 24.599 visits were made to the PED, 4,539 (18.5%) of which concerned adolescents, aged 13 to 17. We excluded 644 accesses for scheduled re-visits, so the total number of visits among adolescents was 3,895. The number adolescent patients arrived at the PED was 3,062, 1,310 females (42.8%), and 1,752 males (57.2%), with a median age of 15 years (IQR 14-16).

Table 1 shows the main characteristics of the adolescents accessing to the PED in the study period.

**Table 1:** Main characteristics of the adolescents accessing the PED.

	**Visits of adolescents at the PED**
Visits, *n* (%)	3,895 (100)
Patients, *n*	3,062
Age in years, median (IQR)	15 (14–16)
Female, *n* (%)	1,310 (42.8)
**Presenting complaints**, ***n*** **(%)**	
Limb trauma	1,362 (35)
Skin rash	270 (6.9)
Abdominal pain	251 (6.4)
Psycho-motor agitation	153 (3.9)
Other	1,859 (47.7)
**Diagnosis at discharge**, ***n*** **(%)**	
Trauma	1764 (45.3)
Organic disease	1511 (38.8)
Mental health problems	249 (6.4)
Functional disease	152 (3.9)
Other	219 (5.6)
**Diagnosis related to adolescents with trauma**, ***n*** **(%)**	
Contusion/sprain	836 (47.3)
Bone fracture	442 (25.0)
Wound	170 (9.6)
Burn	83 (4.7)
Dislocation	21 (1.2)
Others	212 (12)
**Diagnosis related to adolescents with organic diseases**, ***n*** **(%)**	
Upper respiratory infection	324 (21.4)
Otitis media /external	125 (8.3)
Gastroenteritis	118 (7.8)
Skin infections / abscesses	89 (5.9)
Dermatitis /urticaria	78 (5.1)
Dental caries	52 (3.4)
Tonsillitis	37 (2.4)
Conjunctivitis	35 (2.3)
Urinary tract infection	31 (2.0)
Asthma exacerbation	24 (1.6)
Appendicitis	15 (0.9)
Others	583 (38.5)
**Diagnosis related to adolescents with mental health problems**, ***n*** **(%)**	
Anxiety	58 (23.5)
Psychomotor-agitation	43 (17.4)
Behavioral disorder	14 (5.8)
Depression	9 (3.3)
Suicide attempt	6 (2.5)
Psychosis	8 (2.9)
Somatic symptom disorder	6 (2.2)
Other	105 (42)

The majority of adolescents assessed the PED for trauma (45.3%) and for organic diseases (38.8%).

Traumatized subjects received more frequently diagnosis of contusions/sprains and bone fractures. Diagnoses of organic diseases were mainly related to infectious diseases (Table 1).

A mental health problem was diagnosed in 249 adolescents (6.4%), 158 females (63.4%). Table 2 shows the primary symptoms reported by adolescents with these issues and the list of diagnoses related to mental health problems. Adolescents with mental health problems received a high-priority triage code (yellow or red) in 38% of cases. Fifty-four adolescents with mental health problems were hospitalized. They represented 21.7% of visits for mental health problems and 38% of urgent hospital admissions among adolescents in general. Table 2 shows the list of diagnoses related to mental health problems in admitted patients.

**Table 2:** Main characteristic of adolescents accessing the PED for mental health problems.

Visits related to adolescents with mental health problems, *n* (%)	249 (6.4)
Female, *n* (%)	158 (67)
**Symptoms related to adolescents with mental health problems**, ***n*** **(%)**	
Psychomotor agitation	139 (55.8)
Altered state of consciousness	24 (9.6)
Headache	12 (4.8)
Chest pain	11 (4.4)
Dizziness	9 (3.6)
Anorexia	5 (2.0)
Tachycardia	5 (2.0)
Respiratory distress	5 (2.0)
Abdominal pain	5 (2.0)
Other	34 (13.7)
**Diagnosis related to adolescents with mental health problems**, ***n*** **(%)**	
Anxiety	58 (23.5)
Psychomotor-agitation	43 (17.4)
Behavioral disorder	14 (5.8)
Depression	9 (3.3)
Suicide attempt	6 (2.5)
Psychosis	8 (2.9)
Somatic symptom disorder	6 (2.2)
Other	105 (42)
**Diagnosis related to adolescents hospitalized for**	
**mental health problems, *n* (%)**	54 (21.7)
Psychomotor agitation	13 (5.2)
Anxiety	9 (3.6)
Depression	9 (3.6)
Psychosis	8 (3.2)
Suicide attempt	6 (2.4)
Behavioral disorder	4 (1.6)
Eating disorder	3 (1.2)
Other	2 (0.8)

Among adolescents, the visits not related to trauma were 2,131, 249 (12%) of whom reported a diagnosis of a mental health problem.

Table 3 shows the number of urgent presentations and different outcomes of adolescents compared to children presenting to the PED. Notably, despite a similar rate of hospitalization, the adolescents were admitted more frequently to the neuropsychiatric ward (*p* < 0.001).

**Table 3:** Outcomes of adolescents compared to children accessing to the PED.

	**Accesses of adolescents (13–17 yrs)**	**Accesses of children (0–12 yrs)**	***p*-value**
Patients, *n* (%)	4,539 (18.5)	20,060 (81.5)	–
Age in years, mean, standard deviation	15 ± 1.8	6 ± 3.7	–
Female, *n* (%)	1,960 (43.2)	9,513 (47.4)	–
**Triage code**, ***n*** **(%)**
White (not urgent)	840 (18.5)	6,563 (32.7)	<0.001
Green (minor urgencies)	3,183 (70.1)	12,890 (64.3)	<0.001
Yellow (urgent)	511 (11.2)	1,207 (6.0)	<0.001
Red (emergent/resuscitation)	5 (0.1)	44 (0.2)	0.14
**Outcome**, ***n*** **(%)**
Discharge	4,397 (96.4)	19,514 (97.2)	0.13
Hospitalization	142 (3.6)	546 (2.8)	
**Wards of destination in hospitalized patients**, ***n*** **(%)**
Neuropsychiatric	54 (38)	22 (4.0)	<0.001
Pediatric	35 (24.6)	202 (36.9)	0.047
Orthopedic	24 (17)	87 (15.9)	0.81
Surgery	14 (9.8)	101 (18.5)	0.03
Others	15 (11.2)	134 (24.5)	0.02

Patients who received a diagnosis of mental health problems were more likely to make more accesses in the PED, when compared to patients who received trauma, organic and functional diagnosis, median 2 (IQR 1-3, range 1–17) and median 1 (IQR 1-2, range 1–8), respectively. This difference was statistically significant *p* < 0.0001.

## Discussion

This study describes the adolescent's utilization of a tertiary level PED in Italy. As expected, most of the accesses were related to trauma or infective diseases. We found a prevalence of accesses for trauma among adolescents similar to previous studies (7, 8). Nevertheless, this study showed that mental health problems are a leading cause of urgent hospitalization in adolescence. In this series, adolescent patients accounted for nearly a quarter of patients who accessed the PED, and this percentage was in line with that of previous studies (7, 8).

As mentioned, the principal cause of adolescent's access was trauma, but the presentation of mental health problems was considerably high: 6.4% of adolescents received a diagnosis related to mental health, with anxiety and behavioral disorders as the most common diagnoses. Comparing our results with those of a previous study, performed in a similar setting in 1990's, despite possible geographical and social influencing factors, a rise in the prevalence of visits for mental health problems seems realistic, 6.4 vs. 1.6%, respectively (11). Moreover, the diagnosis of mental health problems can be difficult in the PED setting, in particular in patients with physical symptoms but veiling psychological suffering. So that, it is possible that we underestimated the real prevalence of mental health problems in our population. We found that adolescent girls were more prone to present with a mental health problem than male peers, and this data was in line with previous studies (6, 11, 12).

Aside from traumas, among adolescents, 1 in 8 visits was related to a mental health problem, and a third of the hospitalized adolescents were referred to the neuropsychiatric ward. Admission to this ward was significantly more frequent in adolescents than in younger patients accessing the PED (*p* < 0.001).

Evidence shows that near 20% of adolescents present with a mental health problem (13). Patients with these diseases often seek help, with an increasing trend in recent years, in the emergency services (14–18), which for most of them are the first contact with the health care system. Therefore, pediatricians working in the emergency room should be prepared to receive and perform an appropriate investigation in these patients. In order to better evaluate these subjects, specific screening tools have been developed (16), recommending the need for routine assessment of the family and social functioning, substance abuse, emotional and behavioral distress, with particular attention to suicidal thoughts. We want to emphasize that, in our series, nearly 25% of adolescents diagnosed with a mental health problem, presented with physical symptoms such as headache, abdominal pain, chest pain, respiratory distress, or dizziness. Thus, confirming that in adolescence, mental health problems frequently hide behind physical complaints. Besides, in just 1 year, six adolescents presented for a suicide attempt, confirming the high risk of suicide and self-harm in this age group (19).

The few available studies performed in a PED setting focused on adolescents showing that the main reasons for the PED access were injuries or exacerbations of chronic diseases such as asthma or diabetes (7, 8). On the contrary, this study revealed the significant burden of mental health problems in patient's populations, further raising awareness of these disorders in adolescence. In our setting, these diseases are the first cause of urgent hospitalization in this age group and this study could support the promotion of greater investments in the area of emergency psychiatry for adolescents. These data can contribute to highlight the need for an implementation of specially equipped staff and wards to support these patients, across Institutions.

Our study has some limitations. It was a single-center study, referring to a tertiary-level children's hospital in a medium-sized city, so our results cannot be generalized; however, the substantial number of visits recorded strengthened our findings. Our Institution does not have a psychiatric emergency clinic, but we highlight that it has a neuropsychiatric ward. In Italy, many PED don't have the possibility to admit patients with mental health problems to a neuropsychiatric ward because this specific ward is absent in many Institutions. Therefore, we cannot exclude that some patients were referred to our ED aiming to have inpatient psychiatric care. Moreover, among patients with mental health problems, we did not categorized specifically patients with substance abuse. So that, we could not provide data regarding the rate of admissions related to substance abuse.

This was a retrospective study, so we cannot rule out misclassification of patients. In particular, we classified patients in five main diagnostic categories, according to the results of the visit, but without a rigorous codification through the ICD-10 classification. Finally, patients who access our PED are aged 0 to 17 years old, therefore, we cannot provide data for late adolescence, 18 to 25 years of age.

Covid-19 pandemic and the related measures to limit contagion had a strong influence on PEDs utilization (20). Future studies should investigate the influence of social distancing and lockdown measures specifically on adolescent's PED utilization.

In conclusion, this study described the features of a cohort of adolescents accessing the PED for a whole year. It highlighted the remarkable burden of mental health problems in this cohort of patients, showing that it is a leading cause of urgent hospitalization in this age group.

## Data Availability Statement

All data are presented in the manuscript. Any inquiries can be directed to the corresponding author.

## Ethics Statement

The studies involving human participants were reviewed and approved by Institutional Review Board of the Institute for Maternal and Child Health IRCCS Burlo Garofolo, Trieste, Italy. Written informed consent to participate in this study was provided by the participants' legal guardian/next of kin.

## Author Contributions

GC and EB conceived the study. GC and LP designed the study protocol. LP and RG contributed to the acquisition of the data. MG contributed to the statistical analysis of the data and reviewed the final version of the article. GC, AA, LP, and EB wrote down the draft of the article and contributed to the critical revision of the work. All authors read and approved the final manuscript.

## Conflict of Interest

The authors declare that the research was conducted in the absence of any commercial or financial relationships that could be construed as a potential conflict of interest.

## Publisher's Note

All claims expressed in this article are solely those of the authors and do not necessarily represent those of their affiliated organizations, or those of the publisher, the editors and the reviewers. Any product that may be evaluated in this article, or claim that may be made by its manufacturer, is not guaranteed or endorsed by the publisher.

## Abbreviations

PED: pediatric emergency department.
